# Regulatory mechanism of carbohydrate metabolism pathways on oil biosynthesis of oil plant *Symplocos paniculata*

**DOI:** 10.3389/fpls.2025.1452533

**Published:** 2025-02-06

**Authors:** Wenbin Zeng, Beilei Xie, Yunzhu Chen, Jingzhen Chen, Peiwang Li, Lijuan Jiang, Changzhu Li, Qiang Liu, Yan Yang

**Affiliations:** ^1^ College of Life and Environmental Sciences, Central South University of Forestry and Technology, Changsha, China; ^2^ State Key Laboratory of Utilization of Woody Oil Resource, Hunan Academy of Forestry, Changsha, China

**Keywords:** *Symplocos paniculata*, carbohydrate metabolism, oil biosynthesis, transcriptome, regulatory mechanism

## Abstract

The mechanism underlying oil synthesis in oil plant fruits remains elusive, as sugar metabolism provides the essential carbon skeleton without a clear understanding of its intricate workings. The transcriptome and oil and sugar metabolites’ content of *Symplocos paniculate*, an extraordinary oil plant with immense ecological significance, were subjected to a comparative analysis throughout fruit development. The findings unveiled that the impact of sugar metabolism on oil synthesis varied throughout distinct stages of fruit development. Remarkably, during the initial phase of fruit development from 10 to 90 days after flowering (DAF), pivotal genes involved in starch biosynthesis, such as ADP-glucose pyrophosphorylase (*AGP*), starch synthase (*SS*), and starch branching enzyme (*SBE*), facilitated an earlier accumulation of starch within the fruit. Whereas, during the fruit maturation stage (from 90 DAF to 170 DAF), the expression of phosphofructokinase 1 (*PFK-1*), pyruvate kinase *(PK)* and pyruvate dehydrogenase (*PDH*) enzyme genes involved in the glycolysis pathway was significantly upregulated, thereby facilitating a rapid and substantial accumulation of oil. The sugar metabolism activity of *S. paniculata* fruit exerts a crucial influence on the process of oil synthesis, which is highly dependent on the specific developmental stage. These significant discoveries provide potential candidate genes for advanced genetic improvement using molecular biotechnology, thus enhancing both fruit oil production and modifying the composition of fatty acids.

## 1 Introduction

*Symplocos paniculata* (Thunb.) Miq., a member of the *Symplocaceae* family, is a woody oil plant indigenous to China and holds significant ecological and economic importance. The aforementioned statement has been recognized as one of the most auspicious contenders for the production of bioenergy (Liu et al., 2016). A fully matured tree possesses the capacity to yield a plentiful harvest of up to 20 kg of fruit. The entire fruit showcases a 36.6% oil content with a multitude of oil cells predominantly located in the pericarp, thereby determining the optimal utilization of the entire fruit during oil extraction (Liu et al., 2015). The abundant yield and affluent oil content of *S. paniculata* showcase its immense potential for application in biodiesel production, as well as other industrial sectors such as surfactants, soap, and lubricants (Li et al., 2023). Furthermore, *S. paniculata* demonstrates remarkable adaptability across varying temperature zones and soil conditions. It flourishes in desolate, saline, and severely drought-stricken soil (Liu et al., 2015). Moreover, its naturally stunted growth requires minimal management for commercial-scale cultivation. Nevertheless, there have been scant reports on the exploitation and utilization of this species.

The economic value of this woody plant is often determined by the fruit oil content and quality, as it serves as the primary metabolite of *S. paniculata*. Therefore, comprehending the biosynthesis pathway and regulatory mechanisms of oil becomes crucial in order to enhance seed oil content and composition in plants. Many studies have demonstrated that the synthesis of oil is a complex process involving the distribution of carbon sources, which encompasses numerous enzyme genes and their regulated expression in various cellular compartments such as plastids, endoplasmic reticulum, and cytoplasm (Xu and Shanklin, 2016). This intricate process includes pathways for fatty acid biosynthesis, triacylglycerol (TAG) assembly, as well as glycometabolism (starch biosynthesis and metabolism, glycolysis, gluconeogenesis, and citrate cycle) (Lin et al., 2017).

Carbohydrate metabolism plays a pivotal role in seed development, as exemplified by the carbohydrate polymers cellulose and hemicellulose that constitute the major components of cell walls, while starch serves as the primary reserve for seed development (Weichert et al., 2010). Not only do carbohydrates provide fundamental nutrients and energy for seed growth and development, but they also furnish precursors (glycolate pyruvate) for oil biosynthesis derived from carbohydrate decomposition and metabolism (Hill, 2003). The tricarboxylic acid cycle furnishes the ATP required for fat biosynthesis, while Glucose-6-phosphate in Glycolysis pathway serves as the primary carbon source for fatty acid biosynthesis (Liu et al., 2013). The study of developmental gene expression profile of Jatropha (*Jatropha curcas* L.) seeds has underscored the crucial regulatory effects of glucose metabolism, starch metabolism and other carbohydrate metabolic pathways on oil biosynthesis (Jiang et al., 2012). Bourgis et al. have reported that the remarkable increase of oil in oil palm is not only correlated with elevated transcription levels of fatty acid synthetases, but also associated with key enzymes involved in specific plastid carbohydrate metabolism (such as phosphofructose kinase, pyruvate kinase and pyruvate dehydrogenase, etc.) (Bourgis et al., 2011). Furthermore, numerous studies have demonstrated that the manipulation of genes associated with carbohydrate metabolism can significantly enhance oil accumulation in plants’ seeds (Allen et al., 2011).

Unfortunately, the close relationship between carbohydrate metabolism and oil biosynthesis has received little attention (Dunahay et al., 1995). Furthermore, there continues to be a dearth of comprehensive investigations into this intricate correlation. Zhao et al. concluded that in Yellowhorn (*Xanthoceras sorbifolia* Bunge), oil is primarily derived from sugar based on the negative correlation observed between changes in soluble sugar quantity dynamics and oil content during seed development (Zhao et al., 2015). Whereas, previous studies have shown a positive correlation between the dynamic changes in oil and carbohydrate quantities (Kennedy et al., 2011). Furthermore, Borek et al. suggest that the inconsistent correlation observed between carbohydrate metabolism and oil biosynthesis in different plant species’ seeds may be attributed to their genetic backgrounds (Borek et al., 2009). Generally speaking, soluble carbohydrates (primarily sucrose) are synthesized by plant leaves through photosynthesis and subsequently transported to the seeds. The relationship between seeds and leaves is one of sink and source in terms of carbohydrate supply and demand. Sucrose serves as the principal form for translocating carbohydrates from source to sink regions, acting as a physiological precursor for fatty acid biosynthesis in oilseeds (Zhao et al., 2015). Hence, the endeavor to scrutinize the impact of carbohydrate metabolism on oil biosynthesis solely based on their quantity dynamic change correlation is insufficient. The formation of triacylglycerols in developing oilseeds is one of the primary biosynthetic events in the cytoplasm of storage cells but the flow of carbon through different carbohydrate metabolism pathways affecting oil biosynthesis is not fully understood. and whether there is a competition of intermediate precursor utilization among them is still unknown.

In this study, fresh fruits of *S. paniculata* at different developmental stages from the same tree were selected as experimental materials. and aim to (1) Examine the temporal fluctuations in the quantitative relationship between oil and sugars; (2) Investigate functional unigenes encoding the key enzymes associated with carbohydrate metabolism and lipid biosynthesis. (3) Identify functional unigenes through complete transcriptome annotation; (4) Reconstruct carbohydrate metabolism and lipid biosynthesis pathways using identified enzymes; and (5) Elucidate the molecular regulatory mechanisms of carbohydrate metabolism on lipid biosynthesis in *S. paniculata*.

## 2 Materials and methods

### 2.1 Plant materials

The fresh fruits (approximately 100 g) of *S. paniculata* were harvested from accession C3 at the experimental station (28°07’10.38” N, 113°02’53.16”E, and 94.5 m) of Hunan Academy of Forestry in Changsha, Hunan, China every 10 days after flowering (DAF) throughout 2022. The sampling was terminated at 170 DAF due to fruit shedding. The fruit samples were split into two groups: The fruits in one group were subjected to drying at a temperature of 70°C for a duration of three days, followed by oil extraction and subsequent fatty acid analysis, while another group of samples was immediately removed from the mother tree and frozen in liquid nitrogen before being stored at -80°C for sugars determination and transcriptome analysis.

### 2.2 Oils component determination

The oil content of the fruit was determined through meticulous soxhlet extraction. The dried fruits were finely ground into a delicate powder and meticulously weighed (M_1_, g), followed by meticulous extraction using petroleum ether (99.7%) at 60°C for a duration of 8 hours. The residual sample underwent vacuum drying at a temperature of 105°C for a duration of two hours, followed by weighing (M_2_, g), Oil content (%) =(M_1_-M_2_)/M_1_ × 100%. Oil extraction was repeated three times for each fruit development stage.

The saponification of 30 mg of fruit oil was carried out in a 2 mL blending solution (0.5 mol/L), which was prepared by adding 28.1 g of potassium hydroxide (KOH) to one liter of methyl alcohol (99.9%). The resulting saponified fruit oil was separated into layers by the addition of 10 mL each of petroleum ether (99.9%) and deionized water, followed by centrifugation at 3000 r/min for 5 minutes using a refrigerated centrifuge model 3H16RI from Hunan Hexi Instruments Co., Ltd., Chang Sha, China. The fruit oil’s fatty acid components were analyzed using a Nexis GC-2030 gas spectrometer (Shimadzu, Japan), and the resulting supernatant was injected into an Agilent HP-88 column with dimensions of 0.25mm×100m for free fatty acid polyester separation. The Supelco 37 Component FAME Mix was utilized for the identification of the fatty acid components.

### 2.3 Sugars content determination

The sugars, including starch, sucrose, fructose, and glucose, were extracted using the modified methods described in a previous study (Fu et al., 2023). In brief, the fresh fruit tissues were finely powdered, weighed accurately, and incubated with 80% ethanol at a temperature of 80°C for a duration of 2 hours. The resulting supernatant was collected for the quantification of sucrose, fructose, and glucose levels. The pellet underwent digestion using a diluted hydrochloric acid solution (3 mol/L) and subsequently neutralization with sodium hydroxide to facilitate starch determination. The determination of starch, sucrose, fructose, and glucose was executed utilizing corresponding detection reagent kits (Solarbio Science & Technology Co., Ltd, Beijing, China) in strict accordance with the provided protocols. Each measurement was conducted with six replicates to ensure accuracy and reliability.

### 2.4 Microscopic observation

The collected fruits were fixed with FAA fixative (a mixture of formaldehyde, acetic acid, and ethyl alcohol, volume ratio 5:5:90), washed, immersed in a glycerol solution, and subjected to vacuum infiltration to ensure complete penetration of glycerol into the tissue voids of the fruit samples. The frozen microtome was set at a temperature of -30°C for sectioning. Fruit samples were rapidly frozen for 30 seconds, and sections with a thickness of 25 μm were obtained as temporary slides (Wang and Guo, 2021). These slides were then stained with Sudan III and K-I staining solution for 20 minutes and subsequently examined using the Motic digital microscopy system (Er et al., 2000).

### 2.5 Transcriptome sequencing analysis

The total RNA of the fruits from different representative developmental stages was extracted in accordance with the manufacturer’s protocol, utilizing the Spin Column Plant Total RNA Purification kit (Sangon Biotech, China). The cDNA library construction was performed following the operational instructions provided by the mRNA-Seq Sample Preparation Kit (Illumina Biotech, USA). The Illumina HiseqTM 2000 sequencing platform was employed for the purpose of sequencing, while Trinity was utilized to *de novo* assemble the transcriptome in order to acquire the non-redundant Unigene (Grabherr et al., 2011). The high-quality reads were deposited in the National Center for Biotechnology Information (NCBI) Short Read Archive (SRA) database, and their corresponding accession numbers SRA357712 were assigned.

The well-assembled unigenes have been aligned to the following esteemed public protein databases: the Non-redundant (NR) protein database, the Gene Ontology (GO) protein database (Conesa et al., 2005), the Clusters of Orthologous Groups (COGs) protein database (Tatusov et al., 2003), and the Kyoto Encyclopedia of Genes and Genomes (KEGG) protein database (Kanehisa and Goto, 2000). And the expression levels of the unigenes were statistically calculated using fragments per kilobase transcriptome per million mapped reads (FPKM) in accordance with Mortazavi et al.’s methodology from 2008. Subsequently, a hierarchical clustering analysis was conducted based on the expression levels of various unigenes.

### 2.6 qRT-PCR verification

The extraction of fruit RNA and the construction of cDNA were carried out as previously described in section 2.4. Twelve key genes associated with glucose metabolism and lipid anabolism (*DGAT1, LPAAT, PADT, SAD, FATA, ACC, PDH, PFK-1, SS, HK MDH* and *GPI*) were chosen as the target genes. The primers used in the assay were designed by Primer Premier 5.0 which are provided in **Supplementary Table S1**. Each reaction being performed in triplicate.

The qRT-PCR reaction program was conducted as follows: an initial denaturation step at 95°C for 2 minutes, followed by 40 cycles of denaturation at 95°C for 15 seconds, annealing and extension at 60°C for 15 seconds, and a final extension step at 95°C for 15 seconds. The relative gene expression levels were calculated with the comparative cycle threshold method (ΔΔCt), where ΔΔCt = (Ct value of target genes in the test group - Ct value of housekeeping genes in the test group) - (Ct value of target genes in the control group - Ct value of housekeeping genes in the control group). The ΔΔCt value and FPKM value of the target unigene at 10 DAF were normalized to 1, and the data at 90, 130, and 170 DAF in other time points were calculated as multiples of the gene expression levels at 10 DAF (Schmittgen and Livak, 2008).

## 3 Results

### 3.1 The dynamic changes of oil content and components

**Figure 1:**
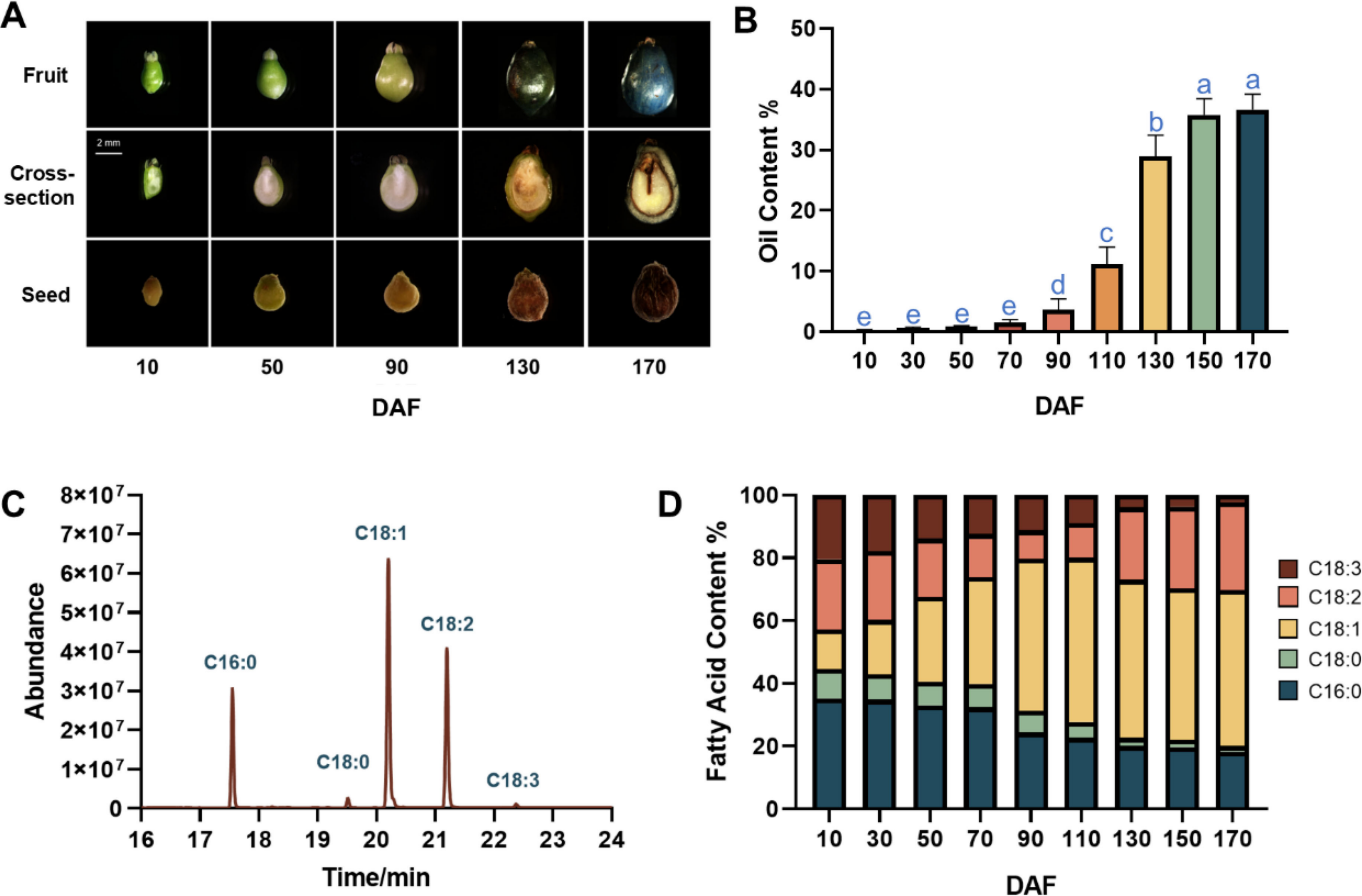
The dynamic changes of oil content and components during *S. paniculata* fruit development. **(A)** the fruit morphological changes, from top to the bottom of figure row illustrates the fruit external morphology, fruit cross-section and seed; **(B)** Dynamic change of fruit oil content, letters indicate differences between developmental stages by Tukey’s honestly significant difference multiple comparison at P < 0.05; **(C)** The gas chromatograph of fruit oil; **(D)** Dynamic change of main fatty acid in fruit oil.

The temporal dynamics of morphological features, oil and fatty acid in the *S. paniculata* fruit were studied throughout its developmental stages. The elongated oval green fruit begins to take shape from 10 days after flowering (DAF), followed by a rapid expansion in volume into yellow-green ovoid fruits up to 90 DAF. Subsequently, there is a slight alteration in the fruit’s volume, accompanied by a transformation of the peel color from yellow-green to shades of yellow-purple and purple, ultimately leading to the fruit’s maturation (**Figure 1A**). While only a small amount of oil (0.23~2.56%) was accumulated in the fruit from 10 to 70 DAF, a remarkable shift occurred during the period between 90 to 140 DAF, with an average daily increase of 0.5% in oil content. Thereafter, the oil content continued to gradually increase until 170 DAF, at which point it reached its maximum fruit oil content of 37% (**Figure 1B**).

The composition of *S. paniculata* fruit oil was predominantly comprised of palmitic acid (C16:0), stearic acid (C18:0), oleic acid (C18:1), linoleic acid (C18:2), and linolenic acid (C18:3) in abundance (**Figure 1C**). The most significant changes entailed a remarkable surge in the proportion of C18:1, skyrocketing from 12% to an impressive 50%, while concurrently witnessing a substantial decline in C16:0 content, plummeting from 35% to a mere 18%. Although alterations in C18:2 content were relatively scarce, they occurred at a quantitatively moderate yet noticeably accelerated pace (27%), surpassing the rate observed during the initial stages of development (22%). The proportions of C18:3 and C18:0 exhibited a continuous decline throughout its development, albeit remaining very low but not negligible in the mature stage (**Figure 1D**).

Collectively, the morphological changes and oil accumulation in *S. paniculata* fruits development can be categorized into three distinct stages.: the Fruit Rapid Expansion (FES) stage (10-90 DAF), the Oil Rapid Accumulation (ORA) stage (90-130 DAF), and the Fruit Dis-Coloration (FDC) stage (130-170 DAF). Fresh fruits at four distinct developmental stages, namely 10, 90, 130 and 170 DAF, were meticulously handpicked for further transcriptomic analysis.

### 3.2 Temporal pattern of sugar metabolites

**Figure 2:**
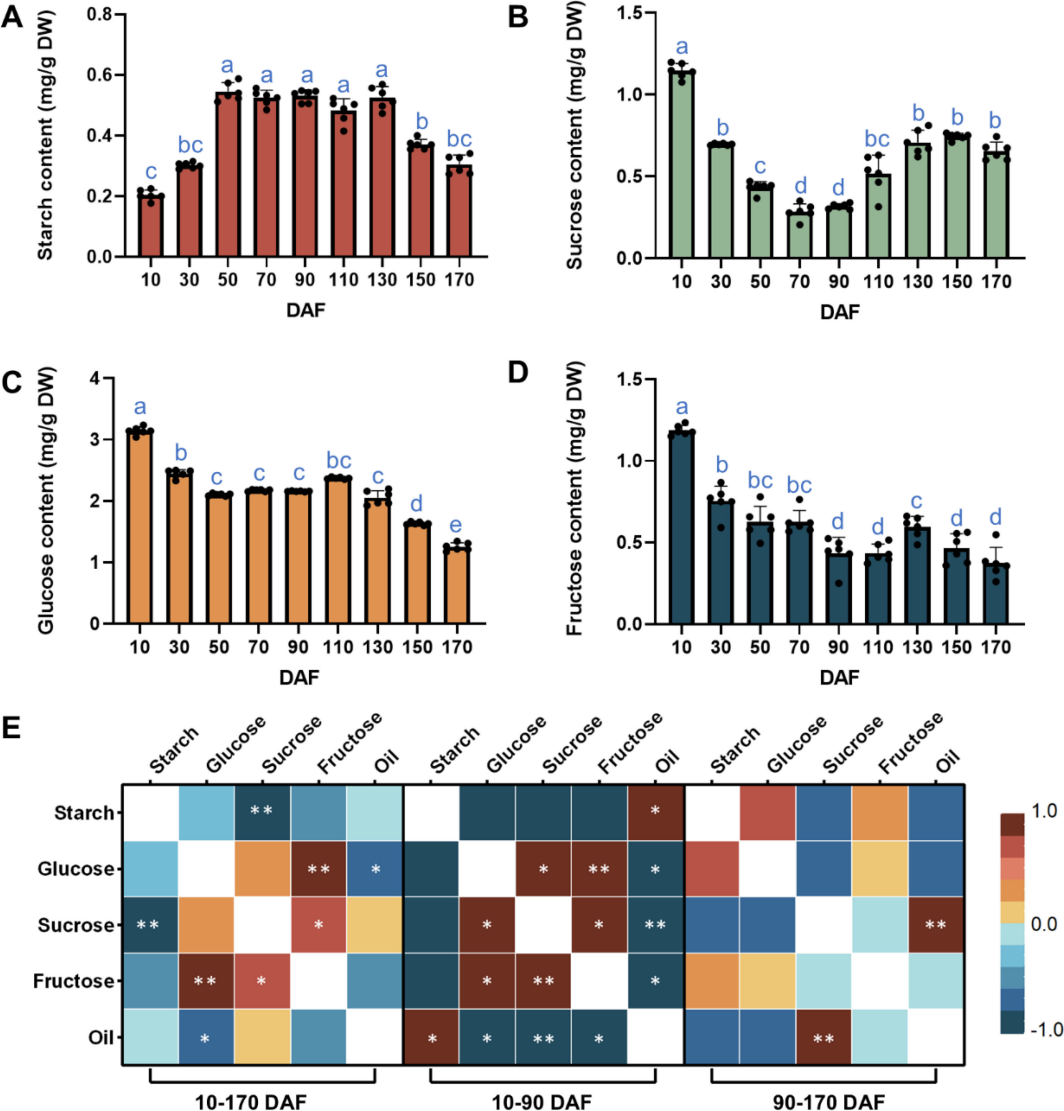
Temporal pattern of sugar metabolites in *S. paniculata* fruit **(A)** Dynamic change of starch content; **(B)** Dynamic change of sucrose content; **(C)** Dynamic change of glucose and content; **(D)** Dynamic change of fructose content; **(E)** Correlation analysis of dynamic changes of main sugar and oil metabolites. Letters indicate differences between developmental stages by Tukey’s honestly significant difference multiple comparison at P < 0.05; ** and * indicates significant correlation at p < 0.01 and p < 0.05 by two-sided test.

The accumulation of oil in fruits is accompanied by the metabolism of carbohydrate substances. The levels of starch, sucrose, glucose, and fructose were determined during the development of *S. paniculata* fruit. The starch content increased progressively during fruit development, reaching a peak of 0.56 mg/g at 50 DAF. Subsequently, it remained relatively stable throughout the ORA stage (90-130 DAF), followed by a rapid decline during the FDC stage. At maturity, the final fruit exhibited a starch content of 0.31 mg/g (**Figure 2A**). The sucrose content exhibited a significant decrease, reaching a minimum of 0.32 mg/g during the FES stage (10-90 DAF), followed by a rapid increase in the fast stage, peaking at 3.346 mg/g at 130 DAF. Throughout the FDC stage (130-170 DAF), there was minimal variation in sucrose content, which remained consistently stable (**Figure 2B**). The glucose and fructose contents exhibited similar patterns, characterized by a significant decrease in the 10-50 DAF content, minimal changes during rapid oil accumulation, and a continuous decline during fruit ripening (**Figures 2C, D**). These findings suggest that the alterations in various sugar contents were distinct and did not align with variations in oil contents.

The correlation between sugar and oil content was further examined. Throughout the entire fruit development stage (10-170 DAF), no significant correlation was observed between oil content and sugar content, except for a significant negative correlation between oil content and glucose. Fructose and glucose exhibited a highly significant positive correlation, while starch and sucrose displayed a highly significant negative correlation. During the 10-90 DAF period of fruit development, there was a positive correlation between oil content and starch content, while a negative correlation was observed with sucrose, glucose, and fructose levels. However, during the critical phase of oil synthesis from 90 to 170 DAF, a highly significant positive correlation between oil and sucrose was observed (**Figure 2E**). No significant correlation was observed with the other three sugars, suggesting that there exists a differential association between sugars and oils at different stages of fruit development.

### 3.3 Temporal pattern of sugar and oil microstructure in fruit cell

**Figure 3:**
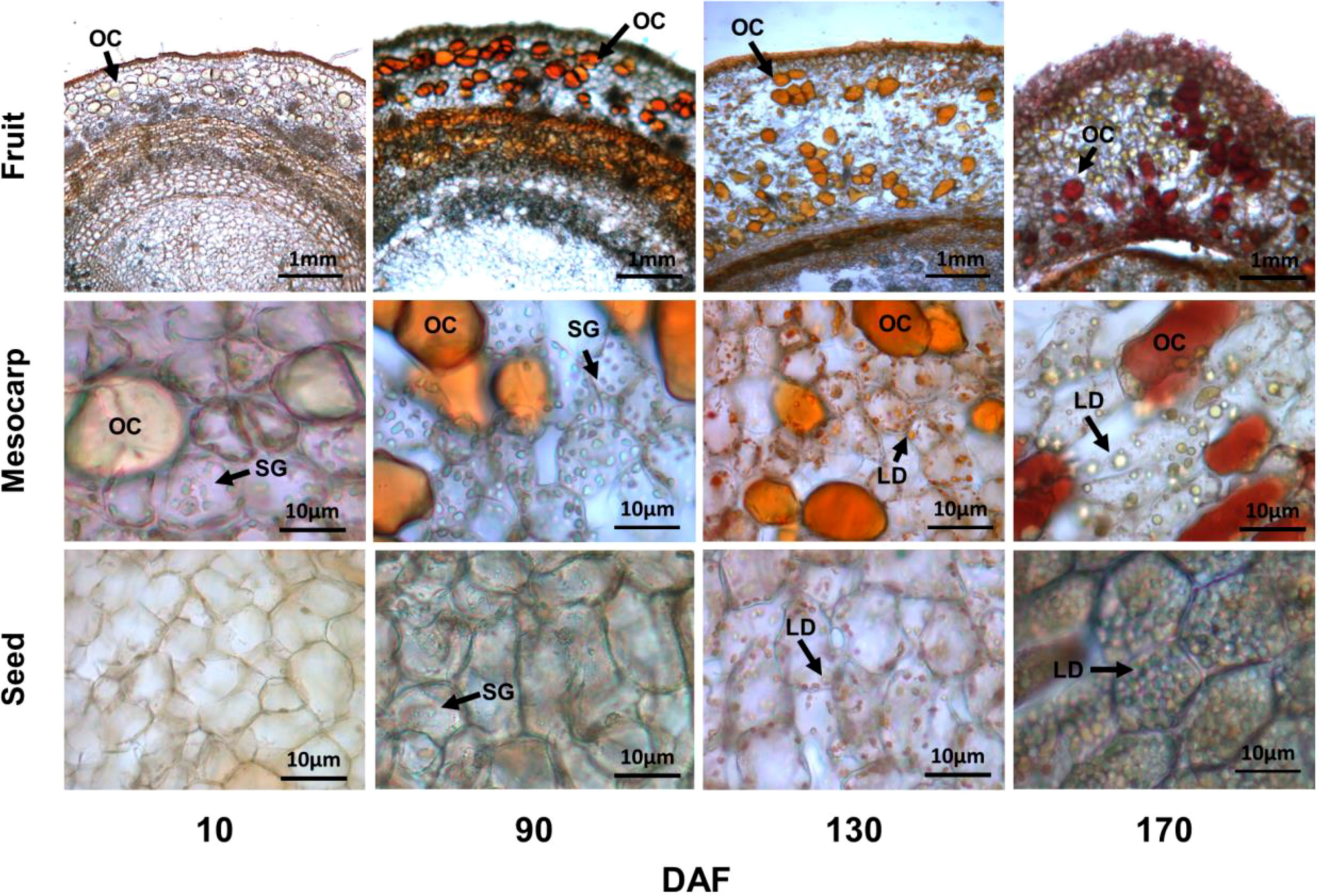
The various developmental stages of fruit cell microstructure. All Sample sections were obtained by frozen sections at 25um, Sudan III (0.5% Dissolved in ethanol solution) staining of oil cells (OC), lipid droplets (LD) and starch granules (SG) by iodine staining (0.5g/L Potassium iodide solution).

Microstructural analysis at different stages of fruit development provides a comprehensive understanding of the oil synthesis process. Microscopic examination revealed that *S. paniculata* fruits (mainly mesocarp and seeds) contained oil, which was stored as oil cells (OC) and cellular lipid droplets (LD). Starch granules (SG) served as the storage structure for starch, while soluble sugars such as sucrose, fructose, and glucose did not exhibit any discernible microstructure (**Figure 3**).

During the initial stage of fruit development (10 DAF), OC were observed in the mesocarp of fruit, scattered throughout the tissue and exhibiting a pale-yellow coloration. The flesh parenchyma cells contained SG, while seed cells lacked both SG and LD. Considering **Figure 1B**, it can be inferred that oil synthesis primarily originated from the mesocarp OC during this early phase of fruit development. Upon rapid fruit oil accumulation (90 DAF), the color of oil cells in the mesocarp transitioned to an orange hue, accompanied by a substantial presence of mature SG in parenchyma cells, which exhibited an oblong or kidney-shaped morphology. Additionally, small SG also emerged within seed cells. This observation was further supported by metabolic content analysis depicted in **Figure 2A**, confirming that fruit starch content reached its peak level.

At the stage of discoloration (130 DAF), a substantial accumulation of orange LD became evident in the parenchyma cells of the mesocarp, accompanied by a gradual reduction in SG. Additionally, an abundance of LD was observed within the seed cells. At 170 days after fruit maturation (DAF), the OC exhibited an orange-red coloration, while the fusion of small and medium LD resulted in the formation of large LD within parenchyma cells. Furthermore, the seed cells contained spherical-shaped LD that completely occupied the cell volume. These findings suggest that oil synthesis in *S. paniculata* primarily occurs in both mesocarp and seed tissues, and the oil synthesis in the mesocarp precedes that in the seed; The synthesis of a substantial quantity of SG precedes the synthesis of oil, whether in the mesocarp or seeds. Additionally, there is an additive effect of OC on fruit’s oil synthesis.

### 3.4 Functional annotation of non-redundant unigenes

**Figure 4:**
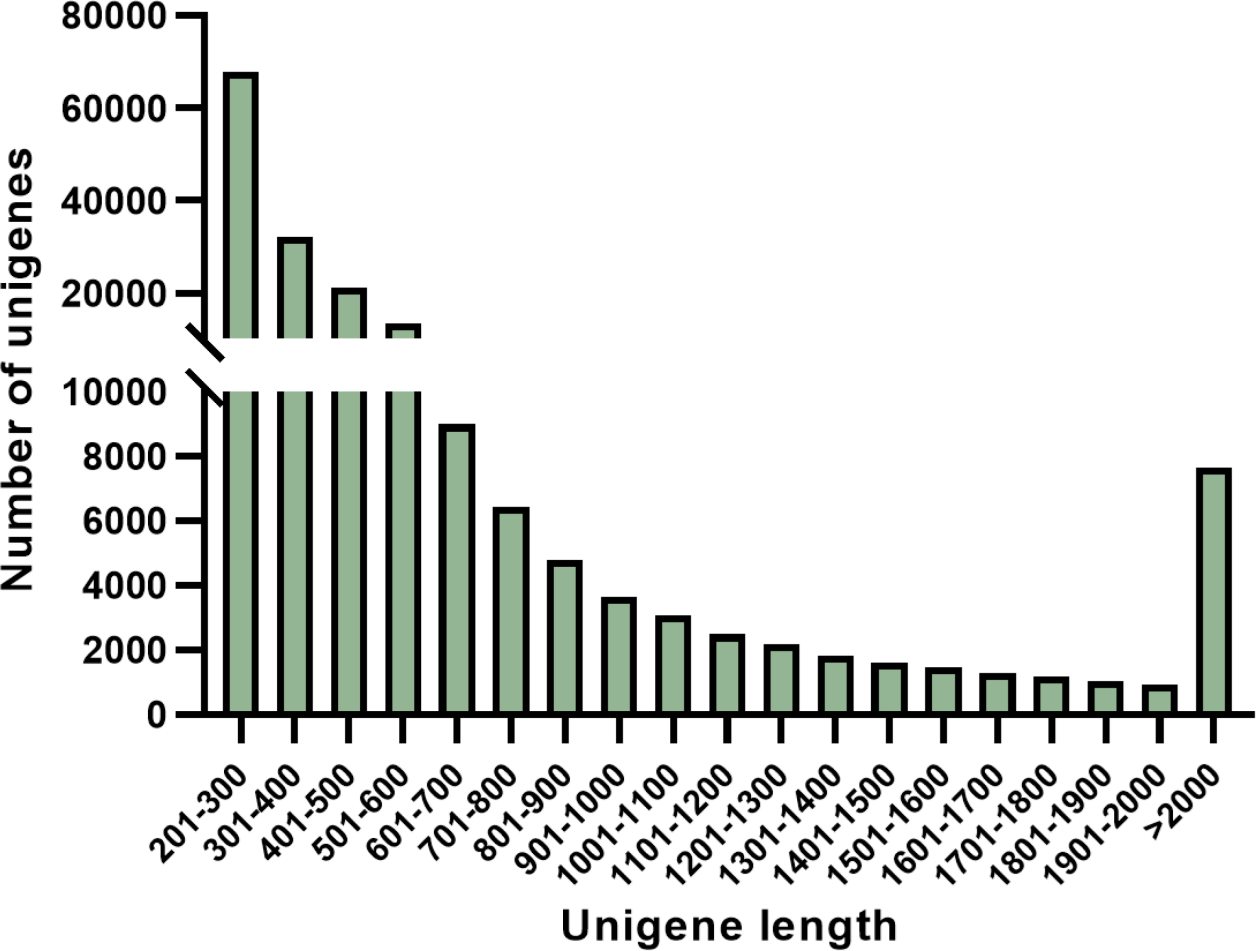
Frequency of *S. paniculata* unigenes.

In this study, a total of approximately 27,827,593 high-quality clean reads (representing 93.86% of the raw reads) were successfully obtained. Following filtration and culling processes, an impressive set of 182,904 non-redundant unigenes were generated. Notably, as the length of unigene sequences increased gradually without any discernible discontinuity (as depicted in **Figure 4**). The aforementioned findings imply that the RNA sequencing was executed with impeccable continuity and exceptional quality, thereby facilitating subsequent analysis.

**Figure 5:**
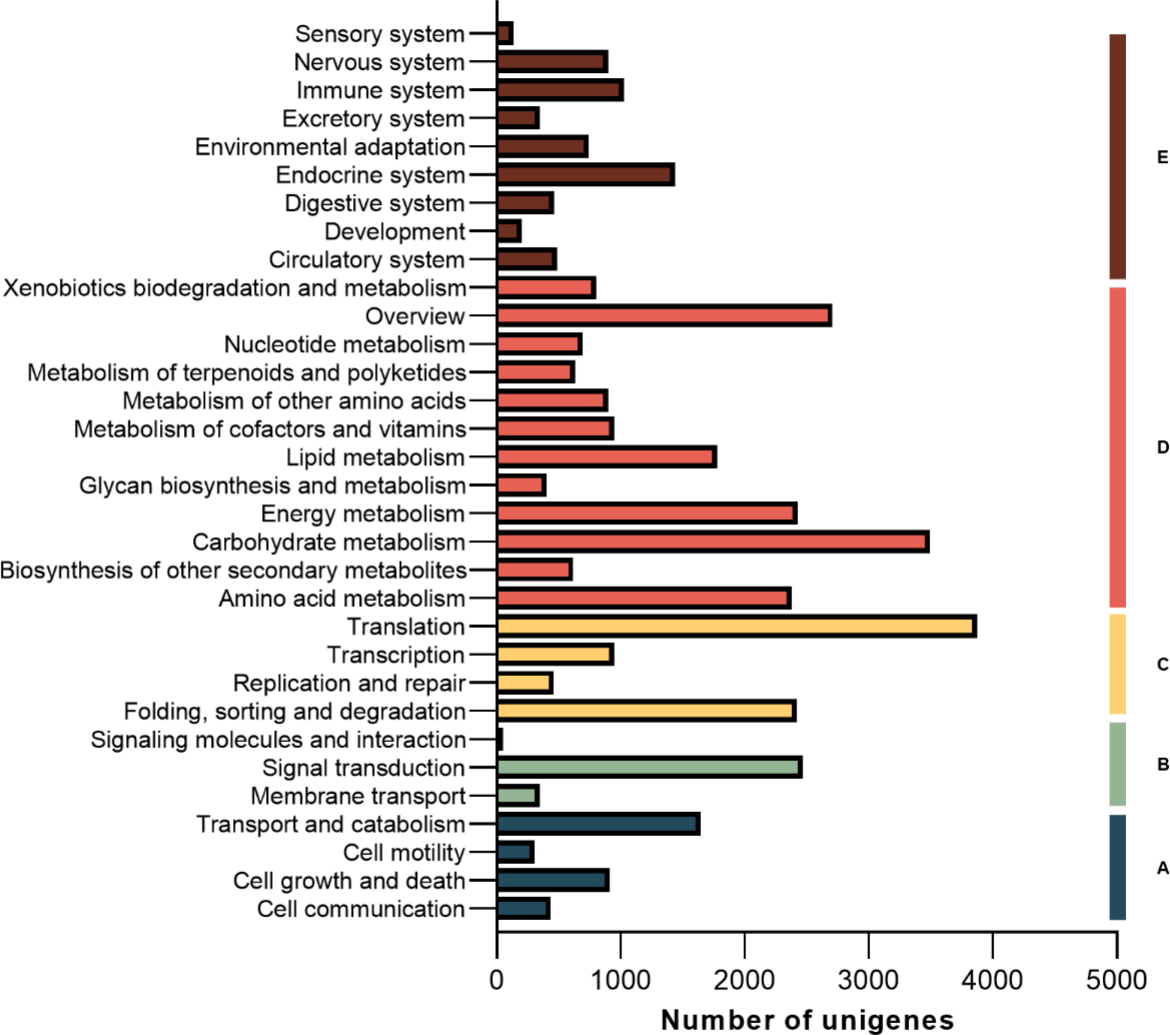
Unigene classifid by KEGG pathways. **(A)** Cellular Processes; **(B)** Environmental Information Processing; **(C)** Genetic Information Processing; **(D)** Metabolism; **(E)** Organismal Systems.

Blast was performed for the unigenes’ functional annotation. The functional annotations of unigenes were obtained by comparing public databases (NR, GO, COG and KEGG). The hit unigenes were meticulously annotated across a total of 32 KEGG pathways. Among these, an impressive count of 1,776 unigenes were precisely mapped to the intricate realm of lipid metabolic pathways, while an astounding number of 3,493 unigenes triumphantly traversed the vast expanse of carbohydrate metabolic pathways (**Figure 5**).

**Figure 6:**
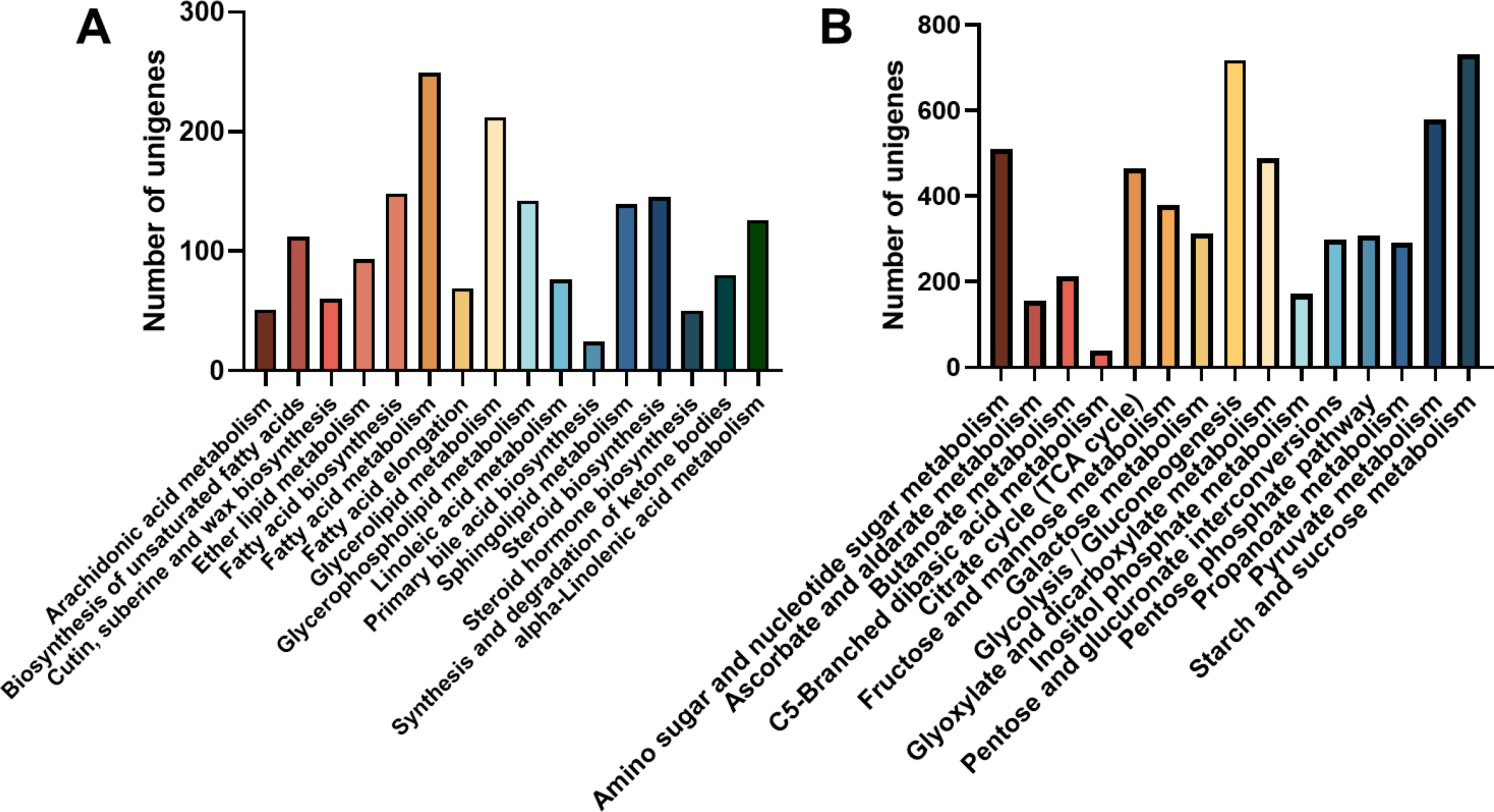
Number of unigene classified to carbohydrate and lipid metabolism and pathway. **(A)** Lipid metabolism pathways; **(B)** Carbohydrate metabolism.

Unigene annotation findings of metabolic pathways related to carbohydrate and lipid metabolism were further analyzed. The pathway of “fatty acid metabolism” boasted the most abundant repertoire of unigenes, totaling 249 in number, this is followed by a substantial number of unigenes in the areas of “glycerolipid metabolism” (212 unigenes), “fatty acid biosynthesis” (148 unigenes), and “glycerophospholipid metabolism” (142 unigenes) (**Figure 6A**). Whereas, among the 15 carbohydrate metabolism pathways, the most frequently annotated unigenes were associated with “Starch and sucrose metabolism (731 unigenes)”, “Glycolysis and Gluconeogenesis (718 unigenes)”, “Pyruvate metabolism (579 unigenes)”, and “Amino sugar and nucleotide glucose metabolism (579 unigenes)”. there are additional pathways related to fatty acid synthesis and metabolism: ‘Pentose phosphate pathway’ has 307 unigenes and ‘Fructose and mannose metabolism’ has 378 unigenes (**Figure 6B**).

**Figure 7:**
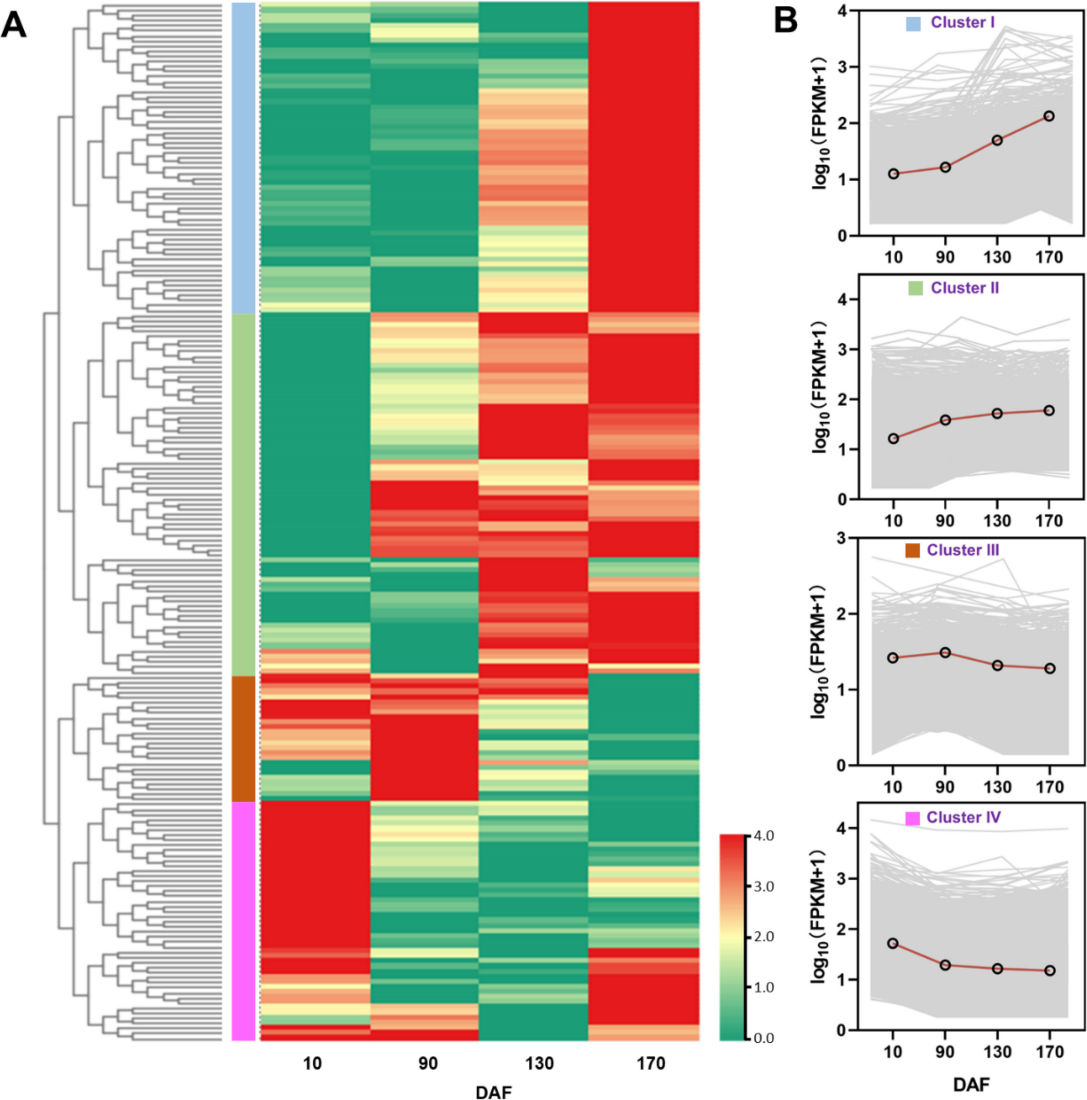
The classification of unigenes associated with carbohydrate and lipid metabolism pathways in *S. paniculata*. **(A)** Clustering tree the unigenes; **(B)** The four different expression trends.

The RPKM values of annotated unigenes associated with carbohydrate and lipid metabolism were utilized to conduct a hierarchical cluster analysis, resulting in the elegant classification of these unigenes into four distinct groups. Unigenes within each cluster exhibited identical or similar expression patterns during fruit oil accumulation stages (**Figure 7A**). The expression pattern of Cluster I and Cluster II exhibited an up-regulated trend, with the FPKM value of the unigenes continuously increasing throughout fruit development. In contrast, Cluster III showed a stable trend during fruit development. Additionally, Cluster IV displayed a down-regulated pattern (**Figure 7B**). The clustering results indicate a relatively distinct expressional profile during the *S. paniculata* fruit development.

### 3.5 The gene expression profiles of sugar and oil metabolism

**Figure 8:**
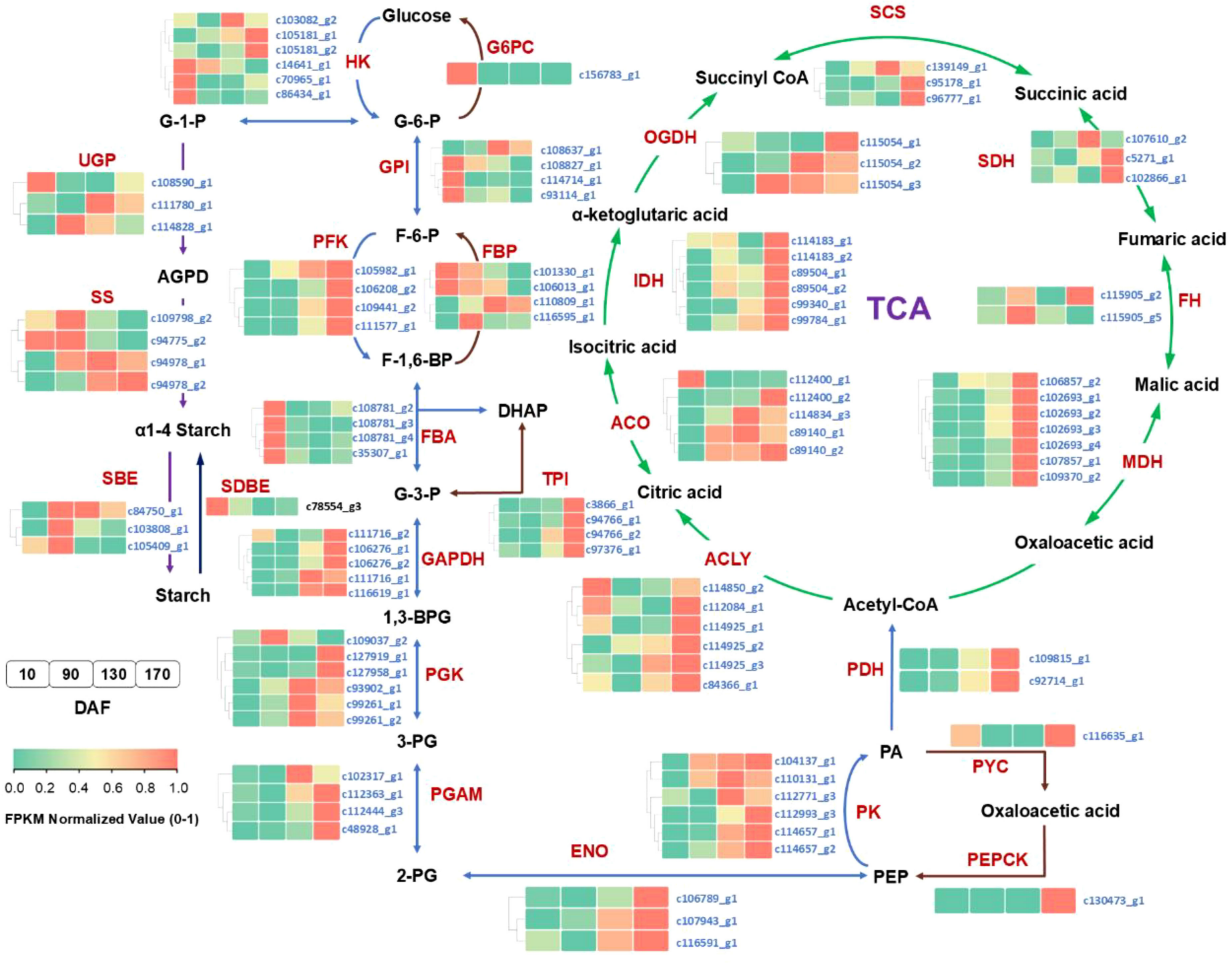
The temporal pattern of key enzyme genes’ expression in sugars metabolism pathways. The blocks in the figure represent the FPKM normalized value at different stages. From left to right: 10, 90, 130 and 170 DAF; FPKM value of all unigenes were normalized to 0 to 1 for comparison. The identified key enzymes involved in carbohydrate metabolism include Hexokinase (HK); hexose phosphate isomerase (GPI); phosphofructokinase 1 (PFK-1); fructose diphosphate aldulase (FBA); triose phosphate isomerase (TPI); glyceraldehyde-3-phosphate dehydrogenase (GAPDH); phosphoglycerate kinase (PGK); phosphoglycerate mutase (PGAM); enolase (ENO); pyruvate kinase (PK); pyruvate carboxylase (PC); phosphoenolpyruvate carboxylase (PEPCK); fructose-1, 6-bisphosphatase (FBP); glucose-6-phosphatase (G6PC); phosphate glucose mutase (PGM); ADP-glucose pyrophosphorylase (AGP); starch synthase (SS); starch branching enzyme (SBE); starch phosphorylase (PYG); starch debranching enzyme (SDBE); pyruvate dehydrogenase (PDH); citrate synthase (ACLY); aconitate hydratase (ACO); isocitrate dehydrogenase (IDH); ketoglutarate dehydrogenase (OGDH); succinyl-coA synthetase (SCS); succinate dehydrogenase (SDH); fumarate esterase (FH); malate dehydrogenase (MDH); sugar substrates are abbreviated: Glucose-6-phosphate (G-6-P); fructose-6-phosphate (F-6-P); fructose-1,6-diphosphate(F-1,6-BP); glyceraldehyde-3-phosphate (G3P); dihydroxyacetone phosphate (DHAP); 1,3-diphosphoglycerate (1,3-BPG); 3-phosphoglycerate (3-PG); 2-phosphoglycerate (2-PG); enolpyruvate (PEP).

**Figure 9:**
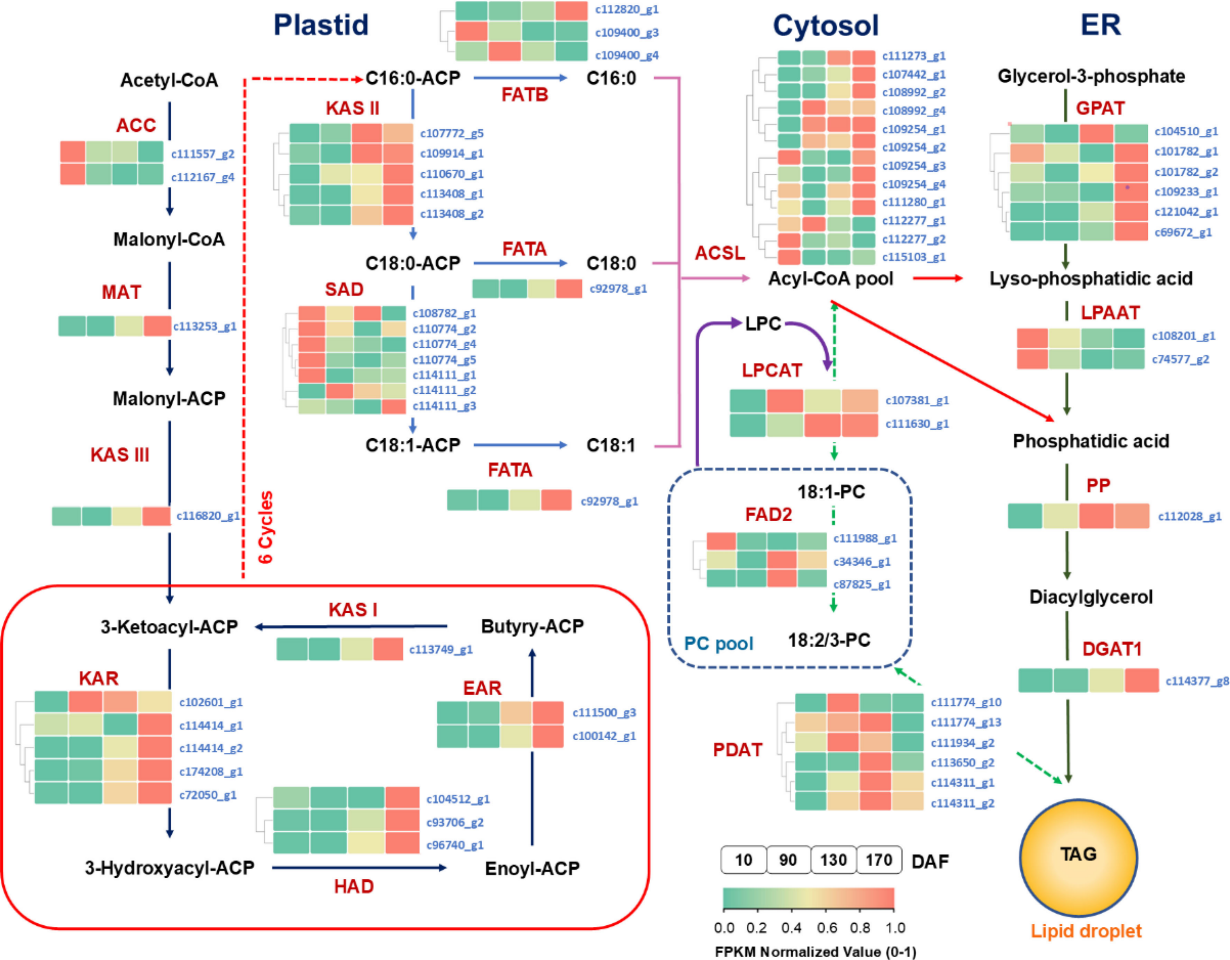
The temporal pattern of key enzyme genes’ expression in oil biosynthesis pathways. The icons close to each enzyme show the results of FPKM normalized value, from left to right were 10, 90, 130 and 170 DAF. FPKM value of all unigenes were normalized to 0 to 1 for comparison. The identified key enzymes involved in lipid metabolism include acetyl-CoA carboxylase carboxyl transferase (ACC); acyl carrier protein (ACP); Malonyl-CoA-ACP transacylase (MAT); 3-Ketoacyl ACP synthase I,II,III (KASI,II,II); 3-Ketoacyl ACP reductase (KAR); 3R-hydroxymyristoyl ACP dehydrase (HAD); enoyl-ACPreductase I (EAR); fatty acyl-ACP thioesterase A (FATA); fatty acyl-ACP thioesterase B (FATB); Stearoyl-ACP desaturase (SAD); long-chain acyl-CoA synthetase (ACSL); fatty acid desaturase (FAD2); glycerol kinase (GK); glycerol-3-phosphate acyltransferase (GPAT); lysophosphatidyl acyltransferase (LPAAT); phosphatidate phosphatase (PP); diacylglycerol O-acyltransferase (DGAT); phospholipid: diacylglycerol acyltransferase (PDAT); lysophosphatidylcholine acyltransferase (LPCAT). Lipid substrates are abbreviated: palmitic acid (16:0); stearic acid (18:0); oleic acid(18:1); linoleic acid(18:2) and linolenic acid (18:3).

The KEGG pathway assignment and functional annotation of the unigenes have revealed the presence of pivotal enzymes involved in carbohydrate and lipid metabolism pathways, which are comprehensively presented in **Supplementary Table S2**. Based on these identified enzymes, an elaborate depiction of the intricate processes governing carbohydrate and lipid metabolism in *S. paniculata* fruit has been meticulously illustrated (**Figures 8**, **9**). In order to gain a comprehensive understanding of the distinct expressional patterns exhibited by specific genes associated with fruit development and oil accumulation, we conducted a comparative analysis of the RPKM values of unigenes across different phases of *S. paniculata*’s fruit oil accumulation.

At the FES stage (10-90 DAF), the oil accumulation exhibited a gradual pace (**Figure 1**), while the pivotal genes involved in glycolysis and tricarboxylic acid cycle (TCA) associated with glucose metabolism displayed low or even down-regulated expression levels. While the enzyme genes responsible for starch biosynthesis, such as ADP-glucose pyrophosphorylase (*AGP*), starch synthase (*SS*), and starch branching enzyme (*SBE*) have shown an up-regulated pattern. Notably, at 90 DAF, their expression levels were observed to be 1.5-fold, 2.4-fold, and 2.1-fold higher than those at 10 DAF.

In conjunction with **Figure 2**, it was observed that the starch content exhibited a rapid surge from 10 to 90 DAF, while the levels of sucrose, fructose, and glucose all experienced a decline. Furthermore, microanalysis in **Figure 3** revealed substantial accumulation of starch granules (SG) within the mesocarp cells at 90 DAF. These findings suggest that during this stage, the primary metabolic activity of the fruit involves the conversion of soluble sugars into starch for storage. At the same time, it was discovered that the genes associated TAG assembly and metabolism were not actively engaged with the exception of acetyl-CoA carboxylase carboxyl transferase (*ACC*) and stearoyl-ACP desaturase (*SAD*). The pivotal gene for fatty acid desaturation, *SAD*, exhibited high expression at 10 DAF to ensure timely C18:0 into C18:1, potentially leading to substantial production of C18:1 during fruit development. However, limited accumulation of C18:0 is the underlying cause. These findings suggest a relative inactivity in genes related to fatty acids or TAG metabolisms. With fruit development, the phosphatidylcholine (PC) pathway revealed a significant up-regulation of lysophosphatidylcholine acyltransferase (*LPCAT*) and phospholipid: diacylglycerol acyltransferase 1 (*PDAT1*) between 10 and 90 DAF, indicating that fruit development may derive a small quantity of oil synthesis from the PC pathway. Additionally, **Figure 3** Microstructure unveiled that oil in fruits at this stage originates from mesocarp oil emphasizing the crucial role of the developmental PC pathway in facilitating mesocarp oil accumulation in Avocado (*Persea americana*) [33]. In conclusion, during FES stage (10-90 DAF), the up-regulation of major starch synthesis genes stimulates starch accumulation while key genes (*LPCAT* and *PDAT1*) in the PC pathway promote modest amounts of oil accumulation within the mesocarp.

Subsequently, during the ORA stage (90-130 DAF), characterized by rapid oil accumulation (**Figure 1**), the genes associated with glycolysis and TCA metabolism, such as phosphofructokinase 1 (*PFK-1*), pyruvate kinase (*PK*), and pyruvate dehydrogenase (*PDH*), exhibited up-regulation. *PFK-1* and *PK* served as pivotal enzymes in the two most crucial irreversible reactions of glycolytic metabolism, facilitating increased conversion of glucose into pyruvate. The up-regulation of *PDH* facilitates the swift conversion of pyruvate into acetyl-coA, as well as malonyl-CoA-ACP transacylase (*MAT*), 3-ketoacyl ACP synthase I/II/III (*KAS I/II/III)*, enoyl-ACP reductase I (*EAR*), 3R-hydroxymyristoyl ACP dehydrase (*HAD*), fatty acyl-ACP thioesterase A (*FATA*) and fatty acid desaturase 2 (*FAD2*) enzymes associated with fatty acid and TAG synthesis. Remarkably enhanced expression levels of glycerol-3-phosphate acyltransferase (*GPAT*), phosphatidate phosphatase (*PP*) and diacylglycerol O-acyltransferase 1 (*DGAT1*) genes indicate that the abundant acetyl-coA during this stage primarily serves oil biosynthesis. Enhanced with **Figure 3**, it was also discovered that an abundance of lipid droplets materialized within the fruit cells during this stage. Furthermore, the gluconeogenesis-associated genes phosphoenolpyruvate carboxylase (*PEPCK*) and pyruvate carboxylase (*PYC*) exhibited down-regulation, while no significant disparity was observed between fructose-1, 6-bisphosphatase (*FBP*) and glucose-6-phosphatase (*G6PC*). The enzyme genes involved in starch synthesis pathway such as SS, SBE, starch phosphorylase (*PYG*), and phosphate glucose mutase (*PGM*) were found to be down-regulated. This observation signifies that gluconeogenesis and starch synthesis remain inactive at this particular stage, thereby ensuring the seamless progression of glycolysis and facilitating substantial oil synthesis.

During the FDC stage (130-170 DAF), oil accumulation continued, and glycolysis-related genes *PFK-1*, glyceraldehyde-3-phosphate dehydrogenase (*GAPDH*), enolase (*ENO*), phosphoglycerate mutase (*PGAM*), *PK* and *PDH* remained highly expressed. Additionally, TCA metabolism-related genes PDH, aconitate hydratase (*ACO*), ketoglutarate dehydrogenase (*OGDH*), citrate synthase (*ACLY*), succinyl-coA synthetase (*SCS*), fumarate esterase (*FH*) and malate dehydrogenase 2 (*MDH2*) exhibited high expression levels. Considering the changes metabolic substance content (**Figure 2**), that active glycolysis and TCA metabolism are likely the primary factors contributing to the simultaneous rapid decline in soluble sugars and starch content during this FDC stage. The genes associated with starch synthesis, namely *SBE* and *SS* down-regulated while the genes related to starch metabolism, *AGP* and *PGM* enzymes, experienced an up-regulation. of starch degradation, thereby signifying that acetyl-coA generated through glycolysis predominantly enters the tricarboxylic acid cycle. Simultaneously, there was a continuous up-regulation in the expression of *KASIII, KAR, EAR, FATA, FATB, FAD2, DGAT1* and other genes involved in fatty acid carbon chain elongation and desaturation pathway. Consequently, leading to a consistent increase in oil content. However, the tricarboxylic acid cycle activity in this stage may also impede oil growth.

### 3.6 qRT-PCR validation for sequencing results

**Figure 10:**
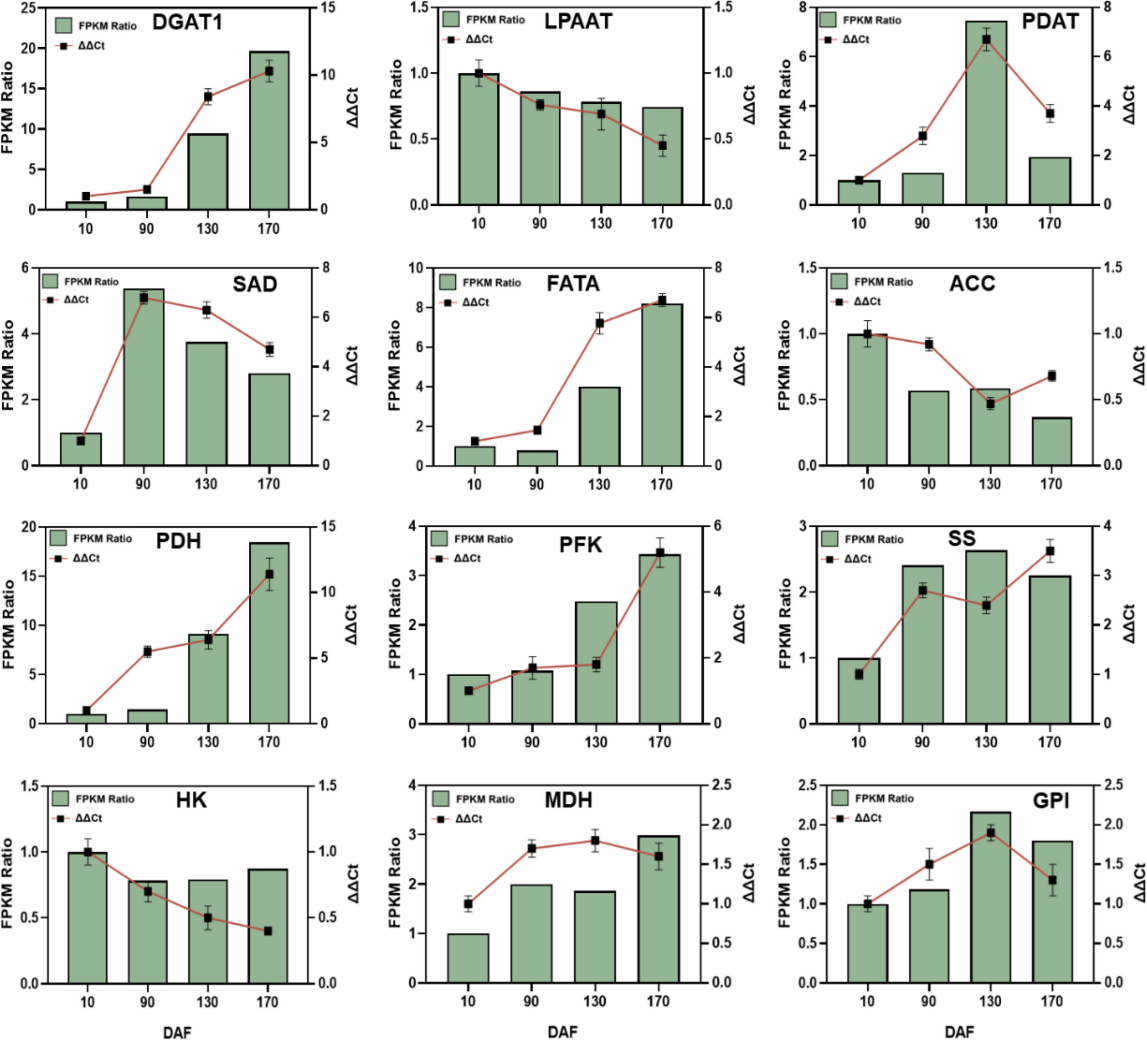
The key genes’ qRT-PCR validation involved in sugar metabolism and oil biosynthesis of *Symplocos paniculate*. The key genes include diacylglycerol O-acyltransferase (DGAT); lysophosphatidyl acyltransferase (LPAAT); phospholipid: diacylglycerol acyltransferase (PDAT); stearoyl-ACP desaturase (SAD); fatty acyl-ACP thioesterase A (FATA); acetyl-CoA carboxylase carboxyl transferase (ACC); pyruvate dehydrogenase (PDH); phosphofructokinase (PFK); starch synthase (SS); Hexokinase (HK); malate dehydrogenase (MDH); and hexose phosphate isomerase (GPI).

To validate the transcriptome sequencing accuracy of *S. paniculata* fruit, this study employed fluorescent quantitative PCR technology to investigate the dynamic changes in relative expression patterns of 12 genes associated with sugar metabolism and fat synthesis (*DGAT1, LPAAT, PADT, SAD, FATA, ACC, PDH, PFK-1, SS, HK, MDH* and *GPI*) (**Figure 10**). The results demonstrated a consistent dynamic change trend in the relative expression of these 12 genes, as determined by qRT-PCR (ΔΔCt), with that observed through transcriptome sequencing and RNA-Seq sequencing (FPKM). These findings further validate the accuracy and reliability of the transcriptome sequencing expression profile, thereby indicating the feasibility of utilizing RNA-Seq technology for differential gene expression pattern analysis in *S. paniculata*.

## 4 Discussion

### 4.1 The influence of sugar metabolism on oil synthesis exhibited diverse patterns across discrete phases of fruit maturation

The sugars present in plant fruits serve as the fundamental basis for oil accumulation, and there exists a close association between carbohydrate metabolism and oil biosynthesis (John and Elspeth, 2003). The investigation on sagebrush (*Salvia* sp*lendens* Ker Gawl.) seed development revealed a negative correlation between soluble sugar content and oil content, suggesting the conversion of soluble sugars into oil during seed maturation. Similar findings were observed in olive (*Olea europaea* L.), where an increase in oil content was accompanied by a decrease in sugar levels, indicating the transformation of sugars into oil. The key principle underlying the transformation of oils by sugars is that glycolysis can supply acetyl-CoA, dihydroacetone phosphate (the synthetic precursor of glycerol 3-phosphate), ATP, and NADH for lipid synthesis. Additionally, the TCA primarily generates ATP while the pentose phosphate pathway mainly provides the necessary NADH for fatty acid synthesis. However, this study revealed stage-specific variations and correlations in the sugars and oils’ contents of *S. paniculata*, indicating the absence of an absolute mutual transformation relationship between these two metabolites. Similar stepwise relationships have been observed during the developmental stages of other oil seeds (Borisjuk et al., 2004). The variations in physiological functions throughout fruit development stages may account for the observed phenomenon. Sugars are not only closely associated with the accumulation of storage substances such as oil and starch (Zhang et al., 2017), but also play a crucial role in providing nutrients and energy for fruit growth and development (Gambetta et al., 2010).

During the FRE stage (10-90 DAF) of *S. paniculata* fruits, starch accumulation exhibited rapid kinetics, while oil accumulation remained minimal. Notably, no significant correlation was observed between oil content and the levels of starch, sucrose, fructose, or glucose. The strong negative correlation observed between sucrose and starch suggests that sucrose is predominantly converted into starch during this stage, a finding further supported by the microscopic examination of mesocarp cell structures (**Figure 3**). Additionally, it should be noted that starch serves as a primary substrate for glycolysis in both plastids and avocado (*Persea americana* Mill.) mesocarp (Kilaru et al., 2015). During the ORA stage (90-130 DAF), oil accumulated rapidly, while starch content remained elevated and sucrose and oil content exhibited significant increases. Furthermore, research has demonstrated that during the intermediate phase of fruit development, a substantial amount of starch accumulates to establish a stable carbon reservoir for oil biosynthesis, thereby facilitating oil synthesis (Vigeolas et al., 2004). The absence of this carbon pool function leads to an increase in soluble sugar content, which in turn limits the rate of sugar transfer in leaves due to high sugar levels. Consequently, during the rapid synthesis of oil, the sustainability of glycolysis process is compromised and there is insufficient carbon source available to maintain optimal activity of related enzymes, resulting in a significant reduction in oil content oilseed rape (*Brassica napus* L.) (Silva et al., 1997). During the FDC period (130-170 DAF), there is a rapid decrease in starch, sucrose, fructose, and glucose levels, which promotes stable oil synthesis through sugar decomposition (Norton and Harris, 1975). Additionally, a study on *X. sorbifolium* fruit revealed a negative correlation between sucrose changes and oil content during the later stages of fruit development, indicating that sucrose decomposition facilitates oil synthesis (Zhang and Liu, 2016).

### 4.2 The pivotal genes within the sugar metabolism pathway of *S. paniculata* fruit exert a profound influence on the synthesis of oil

The synergistic effects between other pathways and lipid anabolic pathways are often overlooked in the study of oil plant biosynthetic pathways. Further investigation is warranted to explore the regulation of enzyme activities related to lipid synthesis, while also considering the contribution of reaction substrates produced by the glucose metabolism pathway. Additionally, prior research has demonstrated a close correlation between glucose metabolism and oil synthesis, with the manipulation of genes associated with glucose metabolism resulting in significant enhancements to seed oil accumulation (Allen et al., 2011). In the investigation of *Arabidopsis thaliana* (L.) Heynh., a model plant, over 800 enzyme genes associated with lipid anabolism were identified, including a substantial number of key genes involved in glycolysis and the TCA pathway (Beisson et al., 2003). The regulatory role of carbon circulation, starch metabolism, and other pathways in oil synthesis was highlighted in the gene expression profile analysis of *Jatropha curcas* seed development (Jiang et al., 2012). The enhancement of oil palm (*Elaeis guineensis* Jacq.) oil is not only associated with the upregulation of fatty acid synthase transcription, but also linked to specific plastid transport factors and key enzymes involved in plastid glucose metabolism, such as phosphofructokinase, pyruvate kinase, and pyruvate dehydrogenase (Bourgis et al., 2011).

In this study, we observed an upregulation in the expression of starch biosynthesis-related enzyme genes during FRE stage (10-90 DAF), including *AGP* (1.5-fold), *SS* (2.4-fold), and *SBE* (2.1-fold), among the key enzymes involved in carbohydrate metabolism pathway. Starch also serves as a primary substrate for glycolysis in plastids, and within the avocado mesocarp, transcripts of genes related to starch synthesis and degradation are abundantly present throughout mesocarp development (Kilaru et al., 2015). Theoretically, starch synthesis reduces the content of monosaccharides which required for glycolysis and would construct an inescapable competition with oil biosynthesis (Bettey and Smith, 1990), but it is found that the synthesis of starch does not restrict but promotes the biosynthesis of oil (Norton and Harris, 1975). Other studies have also found that restricted starch synthesis slowed down or even reduced the accumulation of seed oil. For example, the *PGM* is a key enzyme in starch anabolism, which catalyzed the reversible transformation between glucose-1-phosphate (G-1-P) and glucose-6-phosphate (G-6-P), a noticeable 40% reduction of oil content in Arabidopsis mutant plant with *PGM* gene knocked compare with control (wild type) (Periappuram et al., 2000). Vigeolas et al. reported that the embryo-specific reduction of *AGP* which catalyzed first step of starch synthesis leads to an inhibition of starch synthesis and a delay in oil accumulation in developing seeds of oilseed rape (*Brassica napus*) (Vigeolas et al., 2004). Therefore, these findings suggest that a substantial accumulation of starch occurs during the development of *S. paniculata* fruit, serving as a carbon reservoir for oil synthesis and facilitating enhanced sucrose transport from leaves to fruits, thereby promoting subsequent fruit oil biosynthesis.

A substantial flux through the glycolytic pathway is anticipated to supply the requisite quantities of pyruvate essential for efficient oil synthesis in oil plants. In this investigation, we observed a consistent upregulation of *PFK-1*, *PK*, and *PDH* enzyme genes involved in the glycolysis pathway during the fruit’s ORA stage (90-170 DAF). *PFK-1* and *PK* serve as pivotal enzymes in the two most crucial irreversible reactions within the glycolytic metabolic pathway. The upregulation of *PFK-1* and *PK* facilitates the progression of glycolysis, leading to enhanced conversion of glucose into pyruvate (Chapman and Ohlrogge, 2012). Meanwhile, upregulating *PDH* can expedite the swift transformation of pyruvate into acetyl-CoA, a common precursor for both fatty acid synthesis and TCA cycle, thereby promoting fatty acid biosynthesis (Norton and Harris, 1975). The high oil content in the oil palm fruit is not solely attributed to increased gene expression for enzymes involved in TAG synthesis, but rather exhibits a stronger correlation with plastid glycolytic enzymes (*PFK-1, PK* and *PDH*). The cytoplasmic *PFK-1*, an ATP-dependent enzyme, exhibited a 3.4-fold higher abundance in oil palm mesocarp compared to date palm (a variety primarily accumulating sugar), and demonstrated increased levels during the ripening process of oil palm mesocarp (Bourgis et al., 2011). Therefore, our findings suggest that an upregulation in glycolytic expression plays a pivotal role in facilitating pyruvate provision for the efficient synthesis of fatty acids at high rates.

The TCA serves as a critical link between sugar metabolism and oil biosynthesis. Pyruvate, generated through glycolysis, functions as a shared intermediate in both the oil biosynthesis and TCA cycle pathways. Concurrently, during lipid breakdown, fatty acids undergo β-oxidation while glycerol undergoes the Embden-Meyerhof-Parnas (EMP) pathway to generate acetyl CoA, which subsequently enters the tricarboxylic acid cycle for complete oxidation. In this study, the TCA-related enzyme genes, including *PDH, ACO*, *OGDH, ACLY, IDH, SCS, FH* and *MDH2*, exhibited significantly elevated expression levels during the FDC stage (150-170 DAF). The possible explanation lies in the sharp increase of ripening hormones, such as ethylene, upon fruit maturation, which subsequently impacts the activities of respiratory enzymes and triggers the respiratory climacteric phenomenon (Chapman and Ohlrogge, 2012). The hydrolysis of a substantial quantity of starch and sucrose into glucose for entry into glycolysis results in a rapid reduction in the levels of starch and sucrose. Simultaneously, the upregulation of TCA-related enzyme genes results in enhanced acetyl-CoA production through glycolysis, predominantly fueling the tricarboxylic acid cycle. However, this metabolic shift may impede oil content growth despite its increase during this stage. Previous studies have demonstrated that the addition of hydrogen peroxide (H_2_O_2_) and malonic acid (C_3_H_4_O_4_) during *Chlorella vulgaris* cultivation effectively inhibits intracellular TCA cycle activity, resulting in a significant enhancement of oil content by 34.1% and 28.3%, respectively (Chu et al., 2019). Therefore, modulation of TCA metabolic-related enzyme expression in fruits or reduction of endogenous ethylene levels to attenuate the respiratory climacteric during fruit ripening could potentially serve as a novel approach for augmenting oil synthesis in fruits.

### 4.3 Analysis of the expression patterns of key genes in the lipid synthesis pathway

In the oil synthesis pathway, acetyl-CoA participates in glyceride biosynthesis via soluble proteins or membrane-bound transfer proteins (Yin et al., 2007). The down-regulation of *ACC* was observed in this study, with its expression peaking during the early stage of fruit development. Notably, similar patterns of *ACC* enzyme gene expression were reported in studies on *Prunus sibirica* L (Niu et al., 2015). and *Vernicia fordii* (Hemsl.) Airy Shaw (Chen et al., 2013), corroborating our findings. Relevant studies have demonstrated that the concentration of oleic acid inhibits ACC enzyme activity. Andre et al. (2012). introduced oleic acid into a suspension of European rape liquid culture cells, resulting in the inhibition of ACC enzyme activity in plastid and subsequent reduction in fatty acid synthesis. However, oleic acid constitutes the predominant fatty acid in *S. paniculata* fruit oil. It is worth investigating whether this phenomenon observed during the synthesis of *S. paniculata* fruit oil is associated with the inhibitory effect of oleic acid on ACC enzyme gene expression. Additionally, the transfer of the *ACC* gene into algae for enhancing oil production did not yield any discernible effect (Dunahay et al., 1995). Furthermore, it has been established that the activity of the ACC enzyme can be influenced by the availability of reaction substrates generated through glucose metabolism.

*FATA/B* primarily functions in the hydrolysis of fatty acyl ACP, facilitating the liberation of free fatty acids. Specifically, *FATA* predominantly catalyzes the release of stearoyl ACP and oleoyl ACP (C18:0/1-ACP) to yield stearic acid and oleic acid (C18:0/1), respectively, while FATB mainly mediates the cleavage of palmitoyl ACP (C16:0-ACP) into palmitic acid (C16:0). In this study, we observed a consistent expression of *FATA* from 10 to 90 DAF stage, followed by a subsequent increase from 90 to 170 DAF stage, accompanied by a significant elevation in oleic acid content. While the up-regulation of *FATB* during fruit development stage resulted in a higher palmitic acid content compared to stearic and oleic acid contents at FRE (10-90 DAF) stage. The over-expression of *FATB* in seeds has been shown to result in a four-fold increase in palmitic acid fatty acids in *Arabidopsis* seeds (Dörmann et al., 2000), while disrupting the expression of *FATB* leads to a significant reduction in palmitic acid content, consequently reducing the overall saturated fatty acid content (Bonaventure et al., 2003). Therefore, *FATA/B* would serve as a potential candidate gene for the regulation of fatty acid composition, thereby offering the possibility to genetically modify the relative content of C16 and C18 fatty acids in *S. paniculata* fruits.

The desaturation of fatty acids in the fruit of *S. paniculata* involves two key enzymes. One enzyme, *SAD*, facilitates the conversion of C18:0-ACP to C18:1-ACP within plastids. The other enzyme is present in the cell membrane, endoplasmic reticulum, and chloroplast, and catalyzes the introduction of a second double bond at specific positions along the carbon chain of mono-unsaturated fatty acids. For instance, *FAD2* mediates the desaturation process from C18:1-ACP to generate C18:2-ACP (Tasaka et al., 1996). The expression of the *SAD* gene was observed to be up-regulated by 5-fold at the 10 DAF stage, followed by a sustained and high expression from the 90 to 170 DAF stages. This ensured the timely conversion of stearic acid into oleic acid and facilitated a rapid increase in oleic acid content. The expression of *FAD2* was initially down-regulated and subsequently up-regulated, which played a pivotal role in the biosynthesis of linoleic acid during the late FDC phase (130-170 DAF) of oil accumulation. Modulating the expression of crucial target genes involved in fatty acid desaturation through RNA interference technology is a widely employed approach in genetic engineering for regulating the level of unsaturation in oil plant seeds (Knutzon et al., 1992). In studies on *B. napus* (Knutzon et al., 1992) and *Gossypium hirsutum* L (Liu et al., 2002), silencing the *SAD* gene expression resulted in a significant 38% increase in stearic acid content in seeds of both plants. Similarly, genetic interference targeting the *FAD_2_* gene led to substantial increases in oleic acid content in seeds of *Brassica napus* L. (26%), *Brassica juncea* (L.) Czern. (28%) (Stoutjesdijk et al., 2000), soybean (*Glycine max* (L.) Merr.) (76%) (Wang et al., 2012), and *G. hirsutum* (62%) (Liu et al., 2002). In the context of industrial production and application, biodiesel and edible oil production raw materials have distinct fatty acid composition requirements. An optimal biodiesel should exhibit a high abundance of monounsaturated fatty acids (Wang et al., 2012). Therefore, the incorporation of *SAD* and *FAD2* genes presents a promising avenue for plant genetic modification, enabling precise modulation of *S. paniculata*’s unsaturation degree to cater to diverse production requirements in edible oil and biodiesel sectors.

*GPAT* is the key rate-limiting enzyme that catalyzes the initial step of TAG anabolic pathway, thereby playing a pivotal role in regulating oil synthesis in plants (Shockey et al., 2016). The observed upregulation of the *GPAT* gene expression between 130 and 170 DAF aligns with the trend for rapid increase in oil content during this developmental stage. To enhance oil content in plant varieties, the expression of *GPAT* gene was modulated through genetic engineering techniques (Liu et al., 2012). The safflower plastid *GPAT* gene and *GPAT* gene of *Escherichia coli* were introduced into Arabidopsis plants for targeted expression, resulting in a significant increase of 22% and 15%, respectively, in seed oil content (Jain et al., 2000). Thus, reinforcing the expression of *GPAT* enzyme gene can effectively enhance the oil content of *S. paniculata*.

The *PDAT1* and *DGAT1* genes play pivotal roles in the biosynthesis of TAGs. These two genes exhibit overlapping functions and mutually complement each other [45]. The expression of *PDAT1* was observed to be upregulated at FRE stage (10-90 DAF), followed by downregulation at FDC stage (130-170 DAF). These findings suggest that the PC pathway may contribute to a minor fraction of oil synthesis during early fruit development. The microstructure depicted in **Figure 3** also reveals that the presence of oil in fruits at this stage originates from mesocarp oil cells, highlighting the crucial role of the developmental PC pathway in facilitating mesocarp oil accumulation in Avocado. The *DGAT* gene is more likely to enhance oil content in oil seeds (Kilaru et al., 2015). In this study, we observed a consistent up-regulation of the expression of the *DGAT1* enzyme gene from 90 DAF to 170 DAF. Depletion of the *DGAT1* gene resulted in a significant reduction in oil content in Arabidopsis (*Arabidopsis thaliana*) (Jako et al., 2001), maize *(Zea mays* L*.)* (Zheng et al., 2008), and soybean (*Glycine max*) seeds (Lardizabal et al., 2008).

## 5 Conclusion

**Figure 11:**
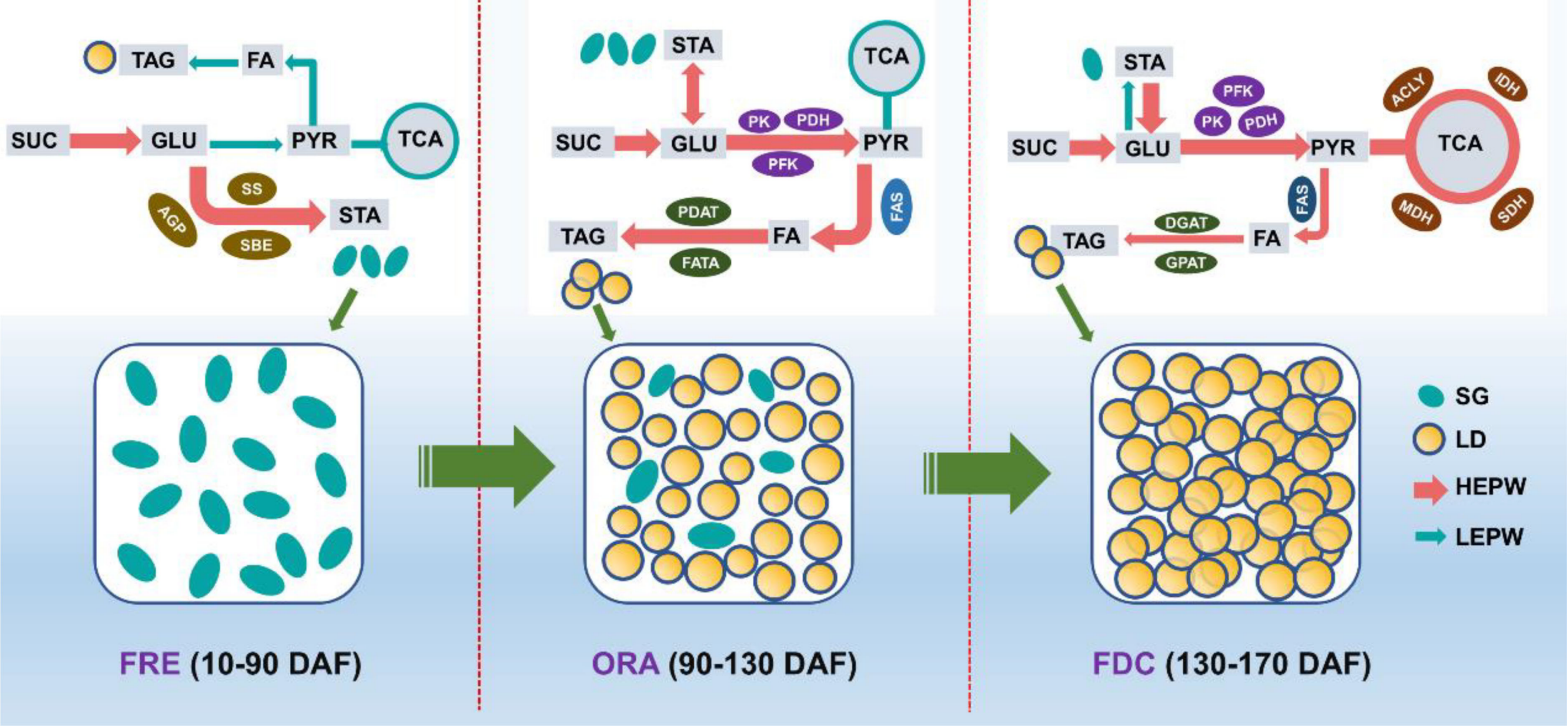
The schematic diagram of the key genes’ temporal expressional patterns involved in the sugar and oil pathways. The width of each arrow is strictly proportional to the level of gene expression in related pathways. HEPW: the high expressional pathway; LEPW: the low expressional pathway; LD: lipid droplets; SG: Starch granules; FES: Fruit rapid expansion stage; ORA: Oil rapid accumulation stage; FDC: Fruit dis-coloration stage; SUC: sucrose; GLU: glucose; PYR: pyruvic acid; FA: fatty acid; TCA: tricarboxylic acid cycle; TAG: triacylglycerol; STA: starch; AGP: ADP-glucose pyrophosphorylase; SS: starch synthase; SBE: starch branching enzyme; PDH: pyruvate dehydrogenase; PK: pyruvate kinase; PFK: phosphofructokinase; FATA: fatty acyl-ACP thioesterase A; DGAT:diacylglycerol O-acyltransferase; PDAT: phospholipid: diacylglycerol acyltransferase; GPAT: glycerol-3-phosphate acyltransferase; MDH: malate dehydrogenase; ACLY: citrate synthase; IDH: isocitrate dehydrogenase; SDH: succinate dehydrogenase.

In this study, we conducted a comprehensive quantitative analysis and employed advanced cell microscopic observation techniques to investigate the dynamic changes in sugar and oil content during different developmental stages of *S. paniculata* fruit. Additionally, we performed a transcriptome analysis to unravel the intricate gene regulatory network underlying these metabolic processes (**Figure 11**). During the FRE stage (10-90 DAF) of *S. paniculata* fruit development, genes *AGP*, *SS*, and *SBE* were upregulated to enhance starch accumulation, a process that involves the conversion of soluble sugars (sucrose, fructose, and glucose) into starch. Concurrently, we observed limited oil accumulation in the mesocarp oil cells, which we attribute in part to the upregulation of the *LPCAT* gene within the PC pathway. Transitioning into the ORA stage (90-130 DAF), we noted high glucose levels serving as a “carbon pool” for oil synthesis. During this period, the upregulation of glycolytic genes PK, PFK-1, and PDH facilitated rapid oil accumulation. Furthermore, genes associated with oil synthesis pathways (Fatty acid biosynthesis and TAG assembly) were generally upregulated, further promoting oil synthesis. In this stage, we observed substantial accumulation of oil bodies in both mesocarp and seed cells. By the FDC stage (130-170 DAF), oil accumulation gradually increased to 36% under the regulation of fatty acid synthesis (*KASIII, KAR, EAR, FATA, FATB, FAD2*) and TAG assembly (*DGAT1*) pathwasys. It is noteworthy that the upregulation of TCA cycle genes during this stage could potentially decelerate the rate of oil accumulation. The study has successfully achieved a comprehensive understanding of lipid synthesis and examined key regulatory genes, thereby laying a solid theoretical foundation for the rational and efficient development of *S. paniculata* resources.

## Data Availability

The datasets presented in this study can be found in online repositories. The names of the repository/repositories and accession number(s) can be found below: https://www.ncbi.nlm.nih.gov/, SRA357712.
