# Supplementary material for: Regulatory mechanism of carbohydrate metabolism pathways on oil biosynthesis of oil plant Symplocos paniculata
Source: Front Plant Sci. 2025 Feb 6;16:1452533. doi: 10.3389/fpls.2025.1452533 (PMC11839820; doi:10.3389/fpls.2025.1452533)
Supplement: Supplementary file 1 [file DataSheet1.docx]

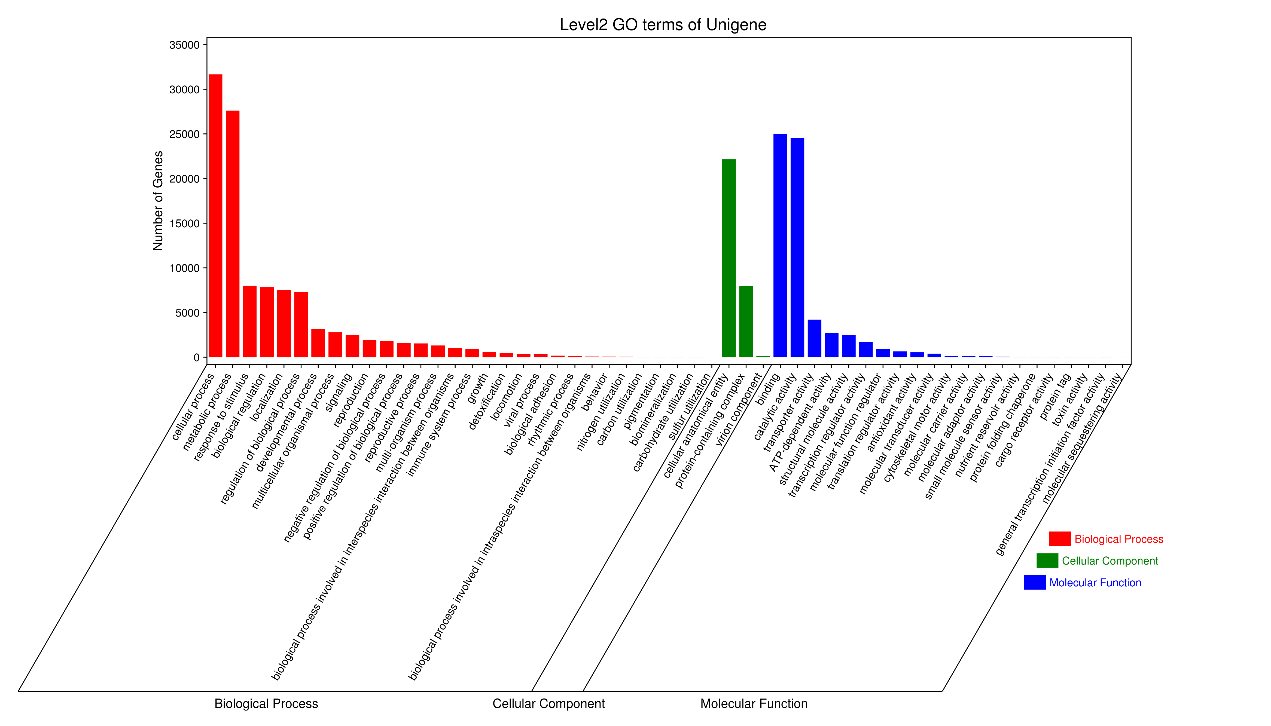


Figure S1 Gene Ontology (GO) categories assigined to the *Symplocos paniculata* unigenes


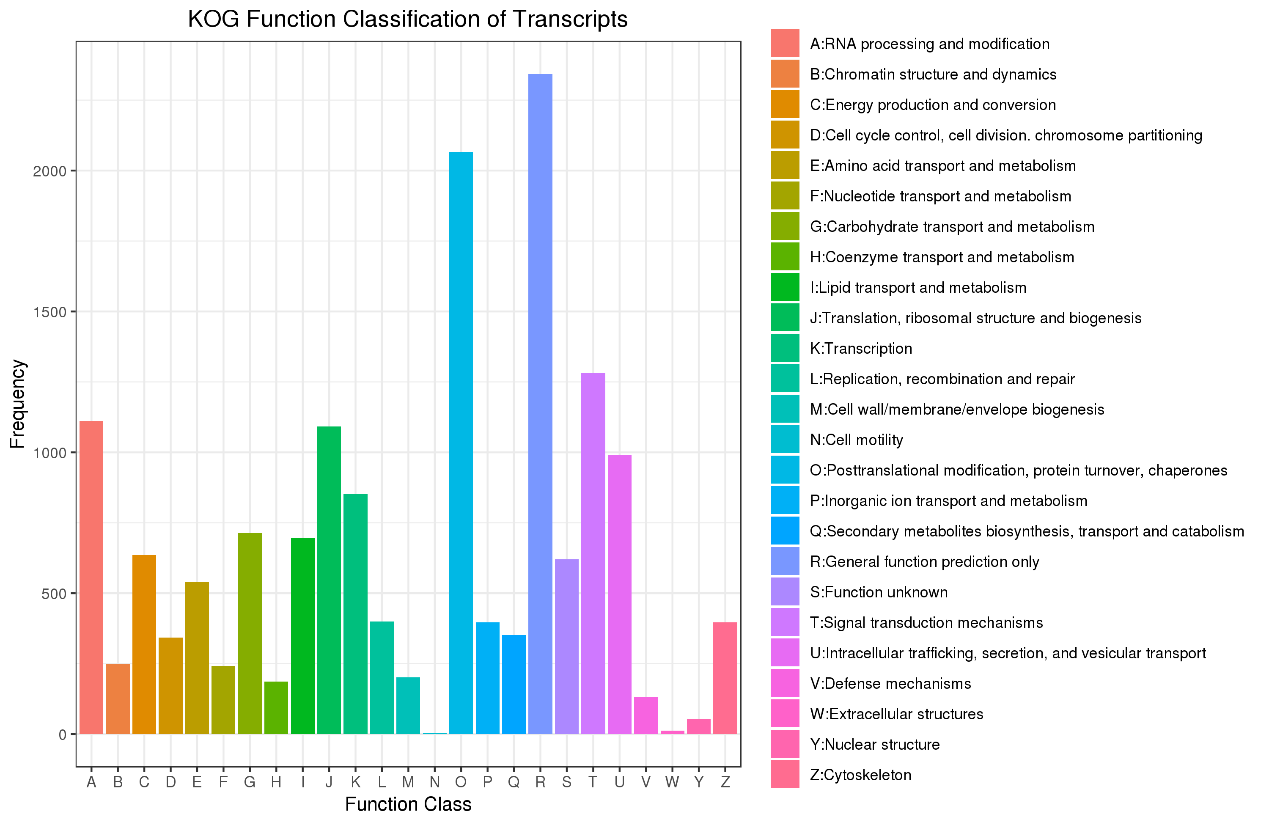


Figure S2 Clusters of Orthologous Groups (COG) categories assigined to the *Symplocos paniculata* unigenes
